# Improved amplification efficiency on stool samples by addition of spermidine and its use for non-invasive detection of colorectal cancer

**DOI:** 10.1186/s12896-015-0148-6

**Published:** 2015-05-29

**Authors:** Jean-Pierre Roperch, Karim Benzekri, Hicham Mansour, Roberto Incitti

**Affiliations:** Profilome, Paris Biotech 24 rue du Faubourg St Jacques, Paris, 75014 France; OncoDiag, Agoranov 96 Bis, Boulevard Raspail, Paris, 75006 France; Centre d’Investigation Clinique (CIC), Henri Mondor Hospital, Créteil, France; King Abdullah University of Science and Technology (KAUST), Bioscience Core Laboratory Research Department, Thuwal, 23955-6900 Saudi Arabia; King Abdullah University of Science and Technology (KAUST), Computational Biology Research Center, Thuwal, 23955-6900 Saudi Arabia

**Keywords:** Spermidine, Colorectal cancer, QM-MSP, Methylated biomarkers

## Abstract

**Background:**

Using quantitative methylation-specific PCR (QM-MSP) is a promising method for colorectal cancer (CRC) diagnosis from stool samples. Difficulty in eliminating PCR inhibitors of this body fluid has been extensively reported. Here, spermidine is presented as PCR facilitator for the detection of stool DNA methylation biomarkers using QM-MSP. We examined its effectiveness with *NPY*, *PENK* and *WIF1*, three biomarkers which we have previously shown to be of relevance to CRC.

**Results:**

We determined an optimal window for the amplification of the *albumin *(*Alb*) gene (100 ng of bisulfite-treated stool DNA added of 1 mM spermidine) at which we report that spermidine acts as a PCR facilitator (AE = 1680%) for SG RT-PCR. We show that the amplification of methylated *PENK*, *NPY* and *WIF1* is considerably facilitated by QM-MSP as measured by an increase of CMI (Cumulative Methylation Index, i.e. the sum of the three methylation values) by a factor of 1.5 to 23 fold in individual samples, and of 10 fold in a pool of five samples.

**Conclusions:**

We contend that spermidine greatly reduces the problems of PCR inhibition in stool samples. This observed feature, after validation on a larger sampling, could be used in the development of stool-based CRC diagnosis tests.

**Electronic supplementary material:**

The online version of this article (doi:10.1186/s12896-015-0148-6) contains supplementary material, which is available to authorized users.

## Background

Colorectal cancer (CRC) is one of the most common forms of cancer in the world [1]. CRC can be cured if diagnosed at early stage using endoscopic examination [2,3], making early non-invasive screening a crucial aim. The development of CRC results from the progressive accumulation of genetic and epigenetic alterations leading to the transformation of normal colonic epithelium to colon adenocarcinoma [4-6]. Fecal-occult blood test (FOBT) is the most widely used method of screening for CRC [7,8]. However, FOBT lacks sensitivity as well as specificity for screening an average risk population. Epigenetic alterations have been found frequently in neoplastic diseases [9,10]. It has been reported that the analysis of DNA methylation carried out in body fluids represents a valuable source for the discovery of cancer biomarkers [11]. Prior studies showed that the hypermethylation can be detected in tumor-derived DNA found in the serum [12-14] and stool [14-17] of patients with CRC. More recently, we proposed a panel of three hypermethylated genes (*NPY*, *PENK* and *WIF1*) as potential biomarkers for the early diagnosis of CRC in tissue and blood samples, based on QM-MSP assay [18]. However, these studies show that the sensitivity of detection must be improved for the application in diagnostic routine. For this reason, the analysis of aberrant methylation in stool DNA might provide a novel strategy for early detection of CRC. The Ahquist’s team demonstrated that blood invasion is more common in advanced stages of CRC where an earlier exfoliation of adenoma and/or tumor cells into the colonic lumen [19]. Moreover, Davies RJ and colleagues showed that the number of colonocytes in the stool following exfoliation from malignant lesions is about 4–5 fold greater than from normal tissue [20] with a mean concentration of 100 ng/g stool, corresponding to 0.01% of the total DNA [21]. However, the composition of feces is highly complex including PCR inhibitors (i.e., bile salts and polysaccharides). It has been reported that the presence of inhibitors can dramatically reduce the sensitivity and amplification efficiency of PCR [22]. As a consequence, consistent extraction of high-quality DNA from fecal samples can be quite challenging, because of the presence of PCR inhibitors that are co-extracted with DNA. Spermidine is a polyamine that has previously been reported to facilitate stool DNA amplification by inhibiting PCR inhibitors [23,24].

Here, we investigated the stimulating effect of spermidine as PCR facilitator for detecting tumor-specific methylated markers in stool DNA.

## Results

### Using the *Alb* gene to test PCR specificity to bisulfite sequencing; checking about absence of interference with PCR ampilication of the *NPY*, *PENK* and *WIF* genes

**Figure 1:**
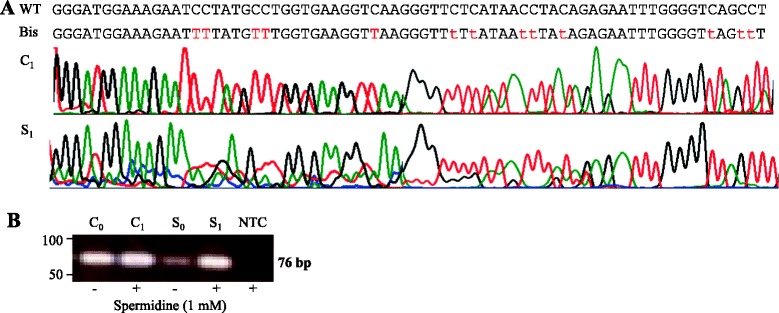
Verification and comparison of PCR amplification products of the *albumin* gene in presence and absence of spermidine. **A**: representative bisulfite sequencing electrophoregram of the *Alb* promoter using SG RT-PCR in presence of 1 mM spermidine from universal methylated human DNA (C_1_) and stool DNA sample (S_1_). All cytosine are converted to thymine noted in red resulting entirely from DNA modification. This follows after sodium bisulfite treatment (Bis) when referring to wild-type (WT) *Alb* gene sequence and **B**: the same PCR products of the *Alb* were analysed by agarose gel electrophoresis and revealed single amplification fragment of the predicted size (76 pb) when spermidine is present (C_1_, S_1_) and absence (C_0_, S_0_); NTC as negative control.

We performed bisulfite sequencing of the entire amplification products in presence and absence of spermidine using nucleic sequences obtained from the *albumin* (*Alb*) gene (data not shown). In Figure 1A, we have represented the sequencing electrophoregram of the *Alb* promoter, as assessed by using SG RT-PCR from C_1_ and S_1_ with 1 mM spermidine. We observed that thymidine are detected instead of cytosine, as expected after DNA bisulfite modification of unmethylated amplicon products, since they correspond to a region of *Alb* which does not contain CpG sites. Those findings indicate that all cytosine are converted to thymine as a result of the DNA modification step being performed successfully and that the spermidine do not interferes in the specificity of PCR. We also verified that spermidine does not interferes the PCR amplification of *NPY*, *PENK* and *WIF1* genes into of CpG rich regions (data not shown).

### Comparing *Alb* PCR products with and without utilization of spermidine

On agarose gel electrophoresis, we observed, in Control (C) and Sample (S) DNA, the 76 bp band confirming the existence of *Alb* gene with or without the presence of spermidine (data not shown). Figure 1B, shows the DNA migration from C (C_0_-C_1_) and S (S_0_-S_1_) with and without 1 mM spermidine addition to the reaction mixture. As expected, we noted a correspondence between band intensities and Ct values with S_0_ (Ct = 28.16) and S_1_ (Ct = 25.11) and not with C_0_ (Ct = 20.05) and C_1_ (Ct = 20.11) (Table 1, 1st serial). The negative template control (NTC) was negative, indicating that it was not nonspecific primer binding or contamination using 1 mM spermidine and also in presence of various concentrations of spermidine, ranging from 1 mM to 10 mM (data not shown).

**Table 1:** **PCR efficiencies in presence and absence of spermidine**

**1st experiment**	**Sperm.**	**Ct**	**∆Ct**	**AE (%)**	**Effects ?**
1st serial					
Control	0	20.05 ± 0.17		100	
	1	20.11 ± 0.02	0.06	96	=
	2	20.77 ± 0.17	0.72	61	-
	3	21.12 ± 0.03	1.07	48	-
	4	21.70 ± 0.32	1.65	32	-
	5	22.66 ± 0.16	2.61	16	-
	10	NA	ND	0	Total inhibition
Sample	0	28.16 ± 0.85		100	
	1	25.11 ± 0.11	−3.06	831	+
	2	25.35 ± 0.03	−2.81	701	+
	3	25.73 ± 0.11	−2.44	541	+
	4	26.53 ± 0.16	−1.63	310	+
	5	28.08 ± 0.13	−0.08	106	+
	10	NA	ND	0	Total inhibition
2nd serial					
Control	0	19.86 ± 0.33		100	
	0.05	19.61 ± 0.10	−0.25	119	+
	0.10	19.78 ± 0.13	−0.09	106	+
	0.50	19.87 ± 0.15	0	100	=
	1	19.91 ± 0.14	0.05	97	=
Sample	0	28.17 ± 0.26		100	
	0.05	26.63 ± 0.49	−1.54	290	+
	0.10	25.98 ± 0.11	−2.19	456	+
	0.50	25.09 ± 0.01	−3.08	843	+
	1	25.22 ± 0.11	−2.95	773	+

Abbreviations: Sperm., spermidine (mM); Ct, mean of cycle threshold value ± standard deviation value; AE, amplification efficiency; NA, non amplification; ND, not determined; =, equal; −, inhibitor; +, facilitator.

The amplification efficiency of the albumin gene at each spermidine concentration points was calculated using 2^-ΔCt^ where ΔCt = (Ct _with spermidine_) – (Ct _without spermidine_). For example in the 1st study, Control DNA with 1 mM of spermidine showing a ΔCt = 0.06, we recovered 96% of the true yield (100%, ΔCt = 0).

### Impact of spermidine on the melting curves of PCR products of *Alb* gene

**Figure 2:**
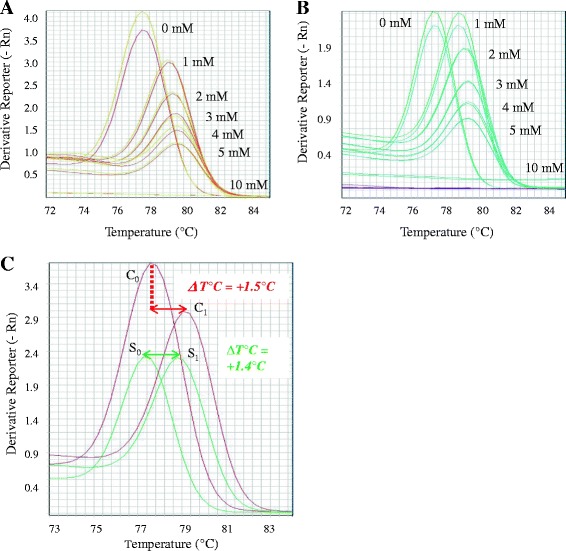
Comparison of melting curves of amplicons of the *Alb* gene using spermidine. We used a mixture of primers to amplify *Alb* gene with an amount of 50 ng of DNA templates at various spermidine concentrations ranging from 0 up to 10 mM. Melting curves of products are shown from C **(A)** and S **(B)**, respectively. In **(C)**, we highlight the melting curves of C and S in presence (C_1_, S_1_) and absence (C_0_, S_0_) of 1 mM spermidine.

In Figure 2 are represented the melting curves of amplicons of the *Alb* gene using spermidine in SG RT-PCR from C (Figure 2A) and S (Figure 2B). We highlight in Figure 2C the melting curves of C and S in presence (C_1_, S_1_) and absence (C_0_, S_0_) of 1 mM spermidine. Both, C_0_ and S_0_ showed a similar temperature of melting (Tm) of 77.7°C and 77.4°C as expected, while for C_1_ and S_1_, we obtained a Tm near 79.2°C (ΔTm = +1.5°C) and 78.9°C (ΔTm = +1.4°C), respectively. (The full results are presented in the supplementary data, Additional file 1: Table S1).

### Checking about PCR inhibition of *Alb* gene when adding spermidine at different concentration

**Figure 3:**
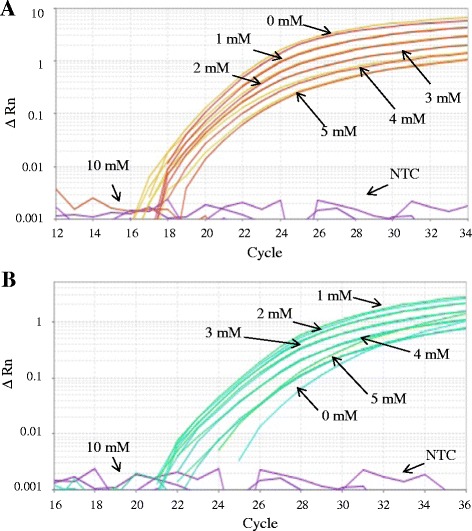
Interference of PCR amplification by spermidine. From 50 ng of DNA, the amplification curves of PCR products of the *Alb* gene using spermidine in SG RT-PCR are shown from C **(A)** and S **(B)**. The spermidine has been used at various concentrations, ranging from 1 mM to 10 mM.

We tested different concentrations of spermidine, ranging from 1 mM to 10 mM (1st serial) and 0.05 mM to 1 mM (2nd serial). In Figure 3 are represented the amplification curves of *Alb* gene (1st serial) illustrating the observed efficiency-Ct shift relationships using spermidine in SG RT-PCR from C (Figure 3A) and S (Figure 3B). In Table 1, the observed result is that low concentrations of spermidine have opposite effects on PCR efficiency of C and S, with a negative effect on C and a positive effect or PCR facilitator on S, (we hypothetise that this effects depend on the purity of DNA samples, assuming C more pure than S) while an excess of spermidine (10 mM) inhibits amplification and this regardless of the nature of DNA. Taken together, those findings suggest that the addition of 1 mM spermidine during PCR cycling might be an optimum for obtaining the highest amplification efficiency on C with for both studies AE = 800 ± 39% (ΔCt = −3.00) of the true yield (AE = 100%, ΔCt = 0 without spermidine) on the detection of *Alb* gene. No significant difference was found on control with AE = 96 ± 0.7% (ΔCt = +0.06). Exceeding 10 mM of spermidine leads to PCR inhibition.

### Varying stool concentration and spermidine concentration

The results are shown in Table 2. They suggest that the 1 mM spermidine together with 100 ng of S DNA provides an optimal amplification efficiency condition, with AE amounting to 1680%.

**Table 2:** **Relationship between spermidine concentration and amount of stool DNA**

	**Sperm.**	**Ct**	**∆Ct**	**AE (%)**
**2nd experiment**				
50 ng	0	28.32 ± 0.15		100
	0.5	25.04 ± 0.11	−3.28	968
	1	25.23 ± 0.17	−3.09	849
	5	28.39 ± 0.21	0.07	95
100 ng	0	27.04 ± 0.16		100
	0.5	23.74 ± 0.12	−3.31	988
	1	22.97 ± 0.24	−4.07	1680
	5	25.49 ± 0.17	−1.55	293
250 ng	0	NA	ND	ND
	0.5	28.67 ± 0.27	ND	ND
	1	23.70 ± 0.37	ND	ND
	5	23.65 ± 0.10	ND	ND
500 ng	0	NA	ND	ND
	0.5	NA	ND	ND
	1	28.00 ± 0.56	ND	ND
	5	26.92 ± 0.30	ND	ND

Abbreviations: Sample, stool DNA pooled; Sperm, spermidine (mM); Ct, mean of cycle threshold value ± standard deviation value; AE, amplification efficiency; NA, non amplification; ND, not determined.

### Measuring promoter methylation of *NPY*, *PENK*, and *WIF1*

The results are shown in Table 3. We evaluated in triplicate assays the performance of QM-MSP to quantify the methylation levels of *NPY*, *PENK*, and *WIF1*. For co-amplifying two methylation-specific DNA targets in real-time, we used the associations of Fam-*Alb*/Vic-*WIF1* and Ned-*NPY*/Vic-*PENK*. QM-MSP were performed from ID1 to ID5 plus S (100 ng) and C (50 ng) with (+) and without (−) the addition of 1 mM spermidine and the methylation percentage was determined by the 2^-∆∆Ct^ method. We summarize the results by using the cumulative methylation index (CMI), which is the sum of the three values. As indicated in Table 3, the results suggest that the addition of spermidine to the PCR mixture allows a sensitive analysis of DNA methylation biomarkers, relative to that not containing spermidine (e.g., on S, the CMI is 4.65 ± 0.67% with (+) and 0.51 ± 0.12 with (−), respectively and reflects a near 10-fold factor of CMI (*P* < 0.01, Student-test)). Furthermore, when we do not observe amplification both in the presence or absence of spermidine, the most likely hypothesis is that the promoter region of this gene is unmethylated (e.g., *NPY* gene from ID 3).

**Table 3:** Quantitative DNA methylation analysis

		**Methylation (%)**			**CMI (%)**
**3rd experiment**		**NPY**	**PENK**	**WIF1**	
ID1	-	0.01 ± 0.01	0.13 ± 0.03	NA	0.14 ± 0.04
	+	0.86 ± 0.26	1.10 ± 0.08	1.22 ± 0.41	3.18 ± 0.75
ID2	-	1.22 ± 0.21	1.31 ± 0.53	0.07 ± 0.18	2.60 ± 0.92
	+	1.86 ± 0.11	1.35 ± 0.09	0.76 ± 0.15	3.97 ± 0.35
ID3	-	NA	NA	0.17 ± 0.07	0.17 ± 0.07
	+	NA	0.10 ± 0.04	1.59 ± 0.22	1.69 ± 0.26
ID4	-	0.17 ± 0.08	0.32 ± 0.10	NA	0.49 ± 0.18
	+	2.33 ± 0.32	1.88 ± 0.44	1.71 ± 0.27	5.65 ± 0.67
ID5	-	0.09 ± 0.08	0.17 ± 0.05	0.07 ± 0.01	0.33 ± 0.14
	+	1.33 ± 0.22	1.44 ± 0.16	1.01 ± 0.07	3.78 ± 0.45
Sample (pooling	-	0.21 ± 0.06	0.27 ± 0.06	0.03 ± 0.03	0.51 ± 0.12
of five patients)	+	1.74 ± 0.30	2.10 ± 0.18	0.81 ± 0.1	4.65 ± 0.67

## Discussion

Detection of precancerous and early-stage CRC is central to improving patient prognosis. Noninvasive colon cancer screening by testing feces for the presence of occult blood still shows poor sensitivity. Consequently, a number of assays for detection of cancer-specific DNA alterations from fecal DNA have been proposed as a new approach for screening and detecting early-stage colon cancer [25-27].

PCR is a powerful technique for the detection of target DNA, but its application to stool specimens is always limited, due to the presence of several components (e.g. bile salts, hemoglobin degradation product, and complex polysaccharides), that are known to often inhibit PCR [28-30]. Problems in eliminating PCR inhibitors from stool specimens have been extensively reported and, for many situations, dilution of inhibited samples proves necessary to provide a rapid and straightforward way of permitting amplification [31]. However, dilution is only possible if the amount of DNA is sufficiently high. Hence, for applications involving low-copy targets and in presence of high background (i.e., bacterial DNA), the dilution solution is often undesirable, and indeed sometimes impossible, due to the further reduction of already reduced target amount [32,33]. Others methods have been used to alleviate the effects of inhibitory substances of PCR, such as the use of separation columns. They present a number of problems too, ranging from reduced DNA yields, leading to decrease DNA targets, to decreased amplification capacity [34-36], and for all these reasons, we have not chosen these methods.

Spermidine, a polyamine compound, has been reported to have a high affinity for plant and stool DNA and could be used as PCR facilitators by its addition to the reaction mixture during the PCR steps [23,24].

In the present study, spermidine concentrations were tested to alleviate PCR inhibition associated with DNA isolated from stool samples. For the first time, we showed that spermidine can act both as an activator on stool DNA or PCR inhibitors on a high purity DNA. Indeed, we showed that, at increasing concentrations, we have 1) on DNA samples extracted from stool: increasing efficiency up to an optimum reached at about 1 mM and then decreasing up to total inhibition and 2) on a set of highly pure DNA: same as 1), but with optimum reached at a much lower concentration, namely 0.05 mM. (We have no explanation for the former.) The hypothesis is that spermidine can block the action of PCR inhibitors (possibly by binding them and or making them more thermolabile, or, alternatively, that by binding DNA at low concentrations it can drastically decrease the action of the PCR inhibitors). The optimum that we observe might be explained by the existence, for each specific sample type, of a concentration threshold above which spermidine no longer blocks PCR inhibitors, may be due to steric effects or saturation, and starts massively binding to DNA, so inhibiting PCR.

Interesting, we observed that the addition of spermidine causes a positive shift of the melting temperature of the stool and control DNA. This observation may be due to the interaction of the spermidine with DNA as it has been described previously by Ahokas and Erkkila [23]. We have determined a range of spermidine concentrations that counteract the PCR inhibitors co-extracted with DNA, so facilitating the amplification efficiency of methylation markers. We applied that finding to assessing the methylation of *NPY*, *PENK*, and *WIF1*, (whose detection is of interest in CRC, as shown in [17]) while maintaining sufficient DNA yield. We showed the advantage of our method in the quantification of methylation values of CRC markers *NPY*, *PENK* and *WIF1*, where, on undiluted stool DNA (100 ng) and by using the QM-MSP in presence of 1 mM spermidine, we globally enhanced detection by a near 10-fold factor, as assessed by summing up the three values. In the future, it would be interesting to evaluate our method with other biomarkers such as Septin 9, which is used as a marker of blood-based methylation requiring improved accuracy for a clinical practise [12].

In summary, we performed a comparative study on the effect of spermidine onto PCR efficiency reporting that spermidine addition is easier and more useful than dilution or purification methods and that it can dramatically improve the quantification of methylation values. We also highlighted a possible mechanism for its action. Our QM-MSP using the presence of 1 mM spermidine and 100 ng of stool DNA could be used as a potential PCR facilitator for stool-based detection of CRC. Our methodology is also a serious candidate for being developed into a robust technology, as it has been optimized with several primer pair and reaction buffer.

## Conclusions

In this study, we present a proof of principle for using spermidine to allow alleviation of the PCR inhibitors frequently encountered in DNA amplification from stool samples. We also demonstrated that spermidine, an inexpensive chemical, is useful for sensitive stool-based detection of methylation-specific markers for CRC tumors using QM-MSP. These results, after corroboration in a large cohort, can lead to the elaboration of a method to be used in clinical practice as a aid in preselecting the patients for colonoscopy.

## Methods

### Human stool samples

We analyzed human stool samples from 5 colonoscopy-negative subjects from the Valihybritest’s collection, registered under the number NCT01270360 (*Clinical Trials.gov*). All of the patients provided informed consent for the research use of their samples.

Use of these samples for this study was approved by the ethical committee of the Val de Marne Paris-Est medical district, registered under code CCP-IDF-IX-11-010.

### Experimental design

This study included three related experiments: the first experiment was designed to study the interference of PCR amplification by spermidine addition at various concentrations ranging from 0.05 mM to 10 mM both 50 ng of universal methylated DNA (Control, C) and 50 ng of stool DNA pooled (Sample, S), each DNA previously modified by sodium bisulfite. The second experiment determined the optimum condition giving the best performance of PCR amplification by modulating both the amount of stool DNA (from 50 ng to 500 ng of modified DNA) and the concentration of spermidine (from 0.5 mM to 5 mM). In these studies, we used spermidine-containing reaction solutions to assess the amplification of the *albumin (Alb)* gene using SYBER Green real-time PCR (SG RT-PCR). In the third experiment, the quantitative methylation-methylation specific PCR (QM-MSP) was performed, with the optimum condition previously defined in 1st and 2nd experiments, to measured the degree of methylation of a three-gene panel consisting of *NPY*, *PENK*, and *WIF1* and calculated the cumulative methylation index (CMI) value of each extract of stool DNA, i.e., ID 1 to ID 5 and Sample.

### Stool DNA isolation and quantification

About 5 g stool were collected from each individual. DNA was isolated from stool samples (200 mg) using the QiAamp DNA stool mini kit (Qiagen) according to the manufacturer’s protocol. DNA concentrations were determined by measurement at 260 nm using BioPhotometer (Eppendorf). Isolated DNA was stored at −20°C.

### Bisulfite modification

1 μg of each DNA (C and S) were modified by sodium bisulfite overnight at 50°C using the EZ DNA Methylation kit (Zymo Research) and eluted in 100 μl of TE buffer (10 mM Tris–HCl (pH 8.0), 1 mM EDTA). Bisulfite treatment converts all unmethylated cytosine residues to uracil (later replicated as thymidine during PCR cycling), while leaving methylcytosines unchanged.

### Primers and probes

For SG RT-PCR, the primers targeting the albumin (*Alb*) gene were reported in Table 1. PCR reactions specific for the *Alb* gene promoter region, which not containing CpG sites. For QM-MSP analysis we used the same primers as those described using SG RT-PCR with in addition the probes targeting *Alb* as Control gene, *NPY*, *PENK* and *WIF1* as CRC-specific genes were reported previously [17] (Additional file 2: Table S2). Primers and probes are designed by Life Technologies company.

### Quantitative real-time PCR analysis

All PCR reactions were carried out in a 96-well reaction plate in a StepOne Plus Real-Time PCR system (Life Technologies) in a final volume of 20 μl. We used the universal methylated human DNA standard (Zymo Research) and positive control (Control) and stool DNA as sample (Sample).

### 1) SG RT-PCR analysis

Modified DNA was analyzed in duplicate by SYBR-Green qPCR master mix (Life Technologies) to determine the optimum concentration of spermidine (Sigma Aldrich), based on albumin amplification product as it accumulates during real-time PCR. The range of spermidine tested is from 50 μM to 10 mM. Five hundred nM of primers (forward and reverse) were also present. The thermal cycling conditions included an initial denaturation at 95°C for 10 min followed by 48 cycles (95°C for 15 s and 60°C for 1 min). The melting curve was determined by heating the PCR product from 60°C to 95°C and monitoring the fluorescence at a transition rate of 0.5°C. The melting temperature or Tm was calculated using the StepOne plus software (Life Technologies), based on the initial fluorescence curve by plotting the negative derivative of fluorescence-reporter (−Rn) over temperature versus temperature (−Rn/T).

### 2) QM-MSP analysis

QM-MSP was performed in triplicate to detect and quantify simultaneously three methylated markers in control and DNA sample using the TaqMan MBG probes technology (Life Technologies). We chose this technique because it allows accurate quantitative assessment of DNA methylation. For each PCR run, a KAPA PROBE master mix (Kapa Biosystems) was prepared, spermidine (1 mM, used as optimum concentration), primers (500 nM) and probes (250 nM) for *Alb*, *WIF1*, *NPY* and *PENK* have been designed (Life Technologies). For co-amplifying two methylation-specific DNA targets in QM-MSP, we used the combinations of Fam/Vic and Ned/Vic fluorophores probes as each probe presents a strong individual spectral intensity with limited overlapping absorption spectra. The PCR cycling parameters were initial denaturation at 95°C for 10 min followed by 95°C for 15 s, 60°C for 1 min, repeated 48 times.

### Bisulfite genomic sequencing

The PCR products of albumin gene were purified before submission to the sequencing process of both strands by using BigDye Terminator Cycle Sequencing kit (Life Technologies) according to the manufacturer’s instructions. The sequence reactions were run and analyzed on an ABI 3100 Genetic Analyzer (Life Technologies). Sequence analyses were performed using ChromasPro software (Technelysium).

### DNA electrophoresis

Five microliters of each reaction were run on conventional 2.5% agarose gel electrophoresis with ethidium bromide (0.5 μg/ml) in TAE buffer (40 mM Tris-Acetate (pH 8.3), 2 mM EDTA). Electrophoresis was done for 2 h in electric field strength of 40 V/cm gel and the DNA was visualized under UV light-transilluminator light (Bio-Rad). The GeneRuler 100 bp DNA ladder (Fermentas) was run on each gel to estimate the size of the PCR products.

### Amplification efficiency measurement

For simplicity, the value (AE) is referred to in the text as amplification efficiency, with any deviations from 100% due to the effects of PCR inhibitors or facilitators in the template DNA (Control and Sample). The Ct of each reaction was yielded by StepOne plus System (Life Technologies) using the amplification-based threshold-determination algorithm; shifts Ct (ΔCt) were measured as the difference between the average Ct template (with spermidine) and the average Ct template (without spermidine). To quantify the inhibition and facilitation effects, we calculated the (AE) value, where AE = 2^-ΔCt^ × 100%.

### Calculation of the methylation percentage

The level of methylation (percentage of methylated reference (PMR)) is quantified according to the calculation of delta-delta Ct (ΔΔCt). We calculate the PMR of each gene by taking 2^-∆∆Ct^ as described see below.

ΔΔ*Ct = [(Ct target, Sample)-(Ct ref, Sample)]-[(Ct target, Control)-(Ct ref, Control)]* where: Ct target, Control = Ct value of gene of interest in control DNA

Ct ref, Control = Ct value of reference gene in control DNA

Ct target, Sample = Ct value of gene of interest in tested sample

Ct ref, Sample = Ct value of reference gene in tested sample

## Additional files

Additional file 1:
**Figure S1.** Effects of spermidine on the temperature melting of the albumin products. Additional file 2:
**Figure S2.** Oligonucleotides.
