# Supplementary material for: Improved amplification efficiency on stool samples by addition of spermidine and its use for non-invasive detection of colorectal cancer
Source: BMC Biotechnol. 2015 May 29;15:41. doi: 10.1186/s12896-015-0148-6 (PMC4446959; doi:10.1186/s12896-015-0148-6)
Supplement: Additional file 1: — Figure S1. Effects of spermidine on the temperature melting of the albumin products. [file 12896_2015_148_MOESM1_ESM.ppt]

## Slide 1
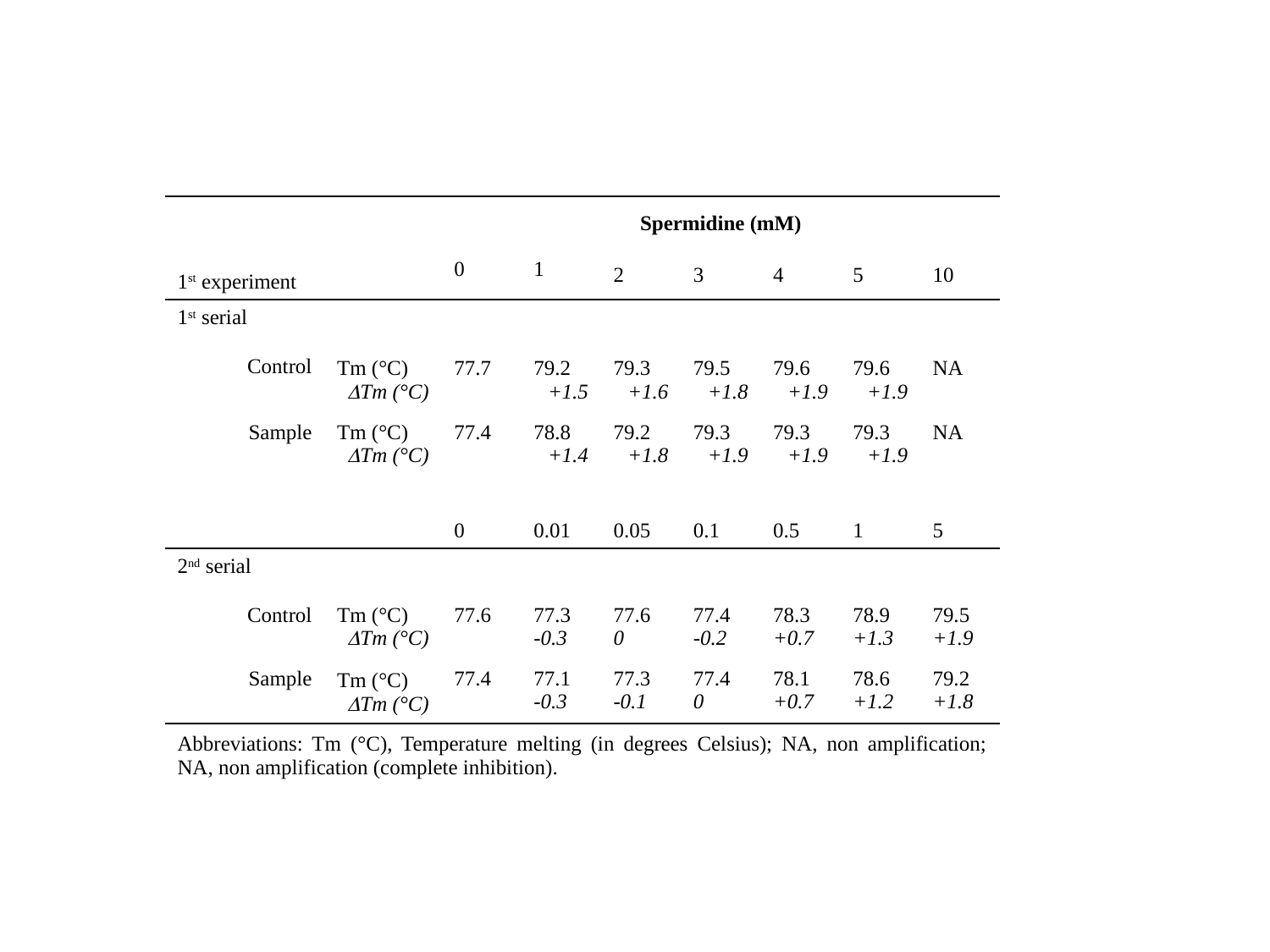

| 1st experiment | | Spermidine (mM) | | | | | | |
| --- | --- | --- | --- | --- | --- | --- | --- | --- |
| | | 0 | 1 | 2 | 3 | 4 | 5 | 10 |
| 1st serial | | | | | | | | |
| Control | Tm (°C) ΔTm (°C) | 77.7 | 79.2 +1.5 | 79.3 +1.6 | 79.5 +1.8 | 79.6 +1.9 | 79.6 +1.9 | NA |
| Sample | Tm (°C) ΔTm (°C) | 77.4 | 78.8 +1.4 | 79.2 +1.8 | 79.3 +1.9 | 79.3 +1.9 | 79.3 +1.9 | NA |
| | | 0 | 0.01 | 0.05 | 0.1 | 0.5 | 1 | 5 |
| 2nd serial | | | | | | | | |
| Control | Tm (°C) ΔTm (°C) | 77.6 | 77.3 -0.3 | 77.6 0 | 77.4 -0.2 | 78.3 +0.7 | 78.9 +1.3 | 79.5 +1.9 |
| Sample | Tm (°C) ΔTm (°C) | 77.4 | 77.1 -0.3 | 77.3 -0.1 | 77.4 0 | 78.1 +0.7 | 78.6 +1.2 | 79.2 +1.8 |
| Abbreviations: Tm (°C), Temperature melting (in degrees Celsius); NA, non amplification; NA, non amplification (complete inhibition). | | | | | | | | |
