# Supplementary material for: Improved amplification efficiency on stool samples by addition of spermidine and its use for non-invasive detection of colorectal cancer
Source: BMC Biotechnol. 2015 May 29;15:41. doi: 10.1186/s12896-015-0148-6 (PMC4446959; doi:10.1186/s12896-015-0148-6)
Supplement: Additional file 2: — Figure S2. Oligonucleotides. [file 12896_2015_148_MOESM2_ESM.ppt]

## Slide 1
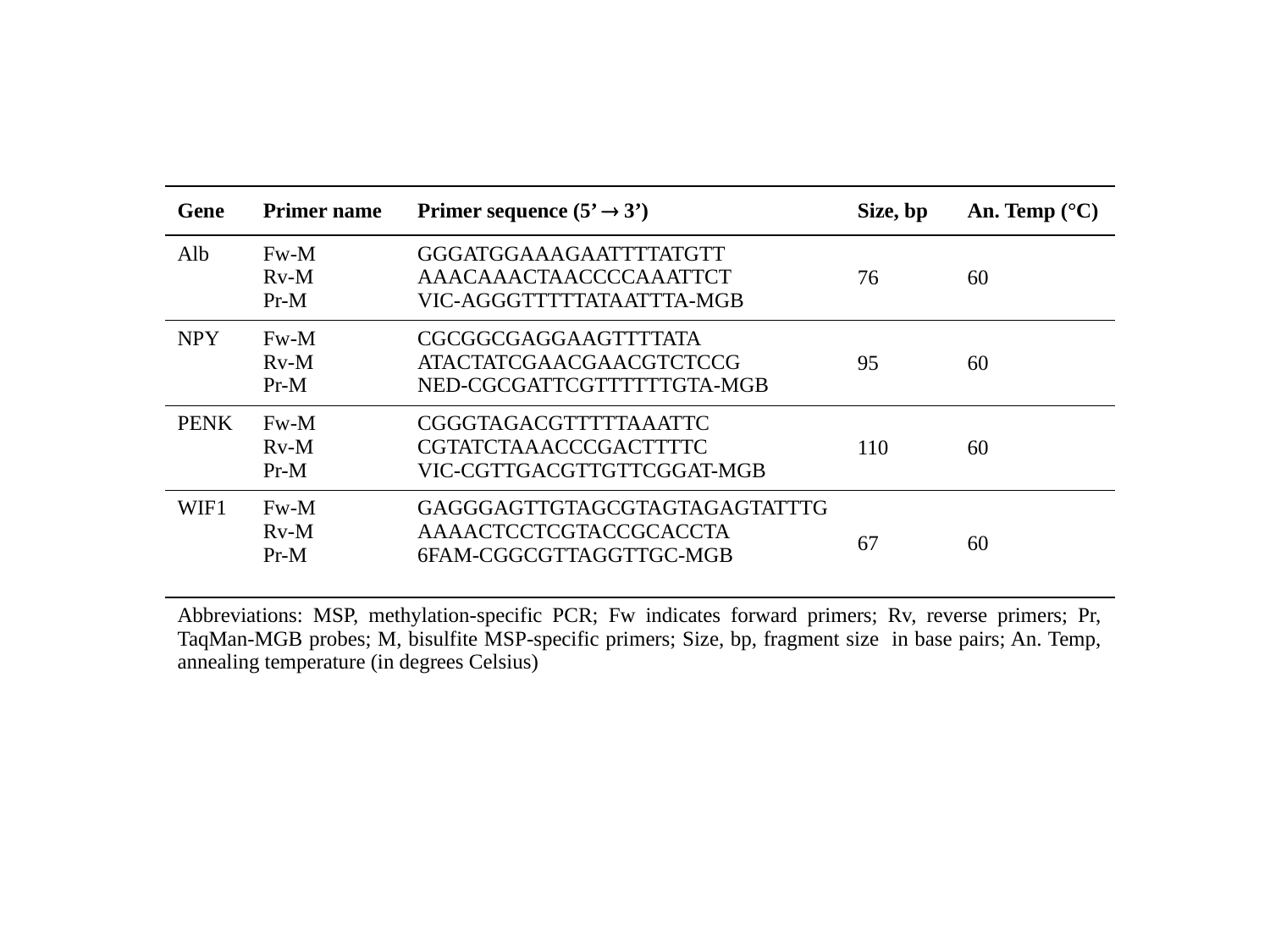

| Gene | Primer name | Primer sequence (5’  3’) | Size, bp | An. Temp (°C) |
| --- | --- | --- | --- | --- |
| Alb | Fw-M Rv-M Pr-M | GGGATGGAAAGAATTTTATGTT AAACAAACTAACCCCAAATTCT VIC-AGGGTTTTTATAATTTA-MGB | 76 | 60 |
| NPY | Fw-M Rv-M Pr-M | CGCGGCGAGGAAGTTTTATA ATACTATCGAACGAACGTCTCCG NED-CGCGATTCGTTTTTTGTA-MGB | 95 | 60 |
| PENK | Fw-M Rv-M Pr-M | CGGGTAGACGTTTTTAAATTC CGTATCTAAACCCGACTTTTC VIC-CGTTGACGTTGTTCGGAT-MGB | 110 | 60 |
| WIF1 | Fw-M Rv-M Pr-M | GAGGGAGTTGTAGCGTAGTAGAGTATTTG AAAACTCCTCGTACCGCACCTA 6FAM-CGGCGTTAGGTTGC-MGB | 67 | 60 |
| Abbreviations: MSP, methylation-specific PCR; Fw indicates forward primers; Rv, reverse primers; Pr, TaqMan-MGB probes; M, bisulfite MSP-specific primers; Size, bp, fragment size in base pairs; An. Temp, annealing temperature (in degrees Celsius) | | | | |
